# Young People Who Meaningfully Improve Are More Likely to Mutually Agree to End Treatment

**DOI:** 10.3389/fpsyg.2021.641770

**Published:** 2021-04-06

**Authors:** Julian Edbrooke-Childs, Luís Costa da Silva, Anja Čuš, Shaun Liverpool, Catarina Pinheiro Mota, Giada Pietrabissa, Thomas Bardsley, Celia M. D. Sales, Randi Ulberg, Jenna Jacob, Nuno Ferreira

**Affiliations:** ^1^Evidence Based Practice Unit, University College London & Anna Freud National Centre for Children and Families, Clinical, Educational and Health Psychology, London, United Kingdom; ^2^Child Outcomes Research Consortium, Anna Freud National Centre for Children and Families, London, United Kingdom; ^3^Department of Child and Adolescent Psychiatry, Medical University of Vienna, Vienna, Austria; ^4^Faculty of Health, Social Care and Medicine, Edge Hill University, Ormskirk, United Kingdom; ^5^Center for Psychology, University of Porto, Porto, Portugal; ^6^Departamento de Educação e Psicologia, University of Trás-os-Montes and Alto Douro, Vila Real, Portugal; ^7^Department of Psychology, Catholic University of Milan, Milan, Italy; ^8^Istituto Auxologico Italiano Istituto di Ricovero e Cura a Carattere Scientifico (IRCCS), Psychology Research Laboratory, Milan, Italy; ^9^Faculty of Psychology and Education Sciences, University of Porto, Porto, Portugal; ^10^Institute of Clinical Medicine, University of Oslo, Oslo, Norway; ^11^Department of Psychiatry, Diakonhjemmet Hospital, Oslo, Norway; ^12^Department of Social Sciences, University of Nicosia, Nicosia, Cyprus

**Keywords:** youth, mental health, outcome, case closure, dropout, meaningful change

## Abstract

**Objective:** Symptom improvement is often examined as an indicator of a good outcome of accessing mental health services. However, there is little evidence of whether symptom improvement is associated with other indicators of a good outcome, such as a mutual agreement to end treatment. The aim of this study was to examine whether young people accessing mental health services who meaningfully improved were more likely to mutually agree to end treatment.

**Methods:** Multilevel multinomial regression analysis controlling for age, gender, ethnicity, and referral source was conducted on *N* = 8,995 episodes of care [Female = 5,469, 61%; *mean*Age = 13.66 (SD = 2.87) years] using anonymised administrative data from young people's mental health services.

**Results:** Compared to young people with no change in mental health difficulties, those showing positive meaningful changes in mental health difficulties were less likely to have case closure due to non-mutual agreement (Odds Ratio or OR = 0.58, 95% Confidence Interval or CI = 0.50–0.61). Similarly, they were less likely to transfer (OR = 0.61, 95% CI = 0.49–0.74) or end treatment for other reasons (OR = 0.59, 95% CI = 0.50–0.70) than by case closure due to mutual agreement.

**Conclusion:** The findings suggest that young people accessing mental health services whose symptoms meaningfully improve are more likely to mutually agree to end treatment, adding to the evidence that symptom improvement may be appropriate to examine as an indicator of a good outcome of accessing mental health services.

## Introduction

Worldwide, poor mental well-being of young people (YP) has been recognized as being a key challenge to be addressed (Camilletti, 2018). Prevalence data estimates that rates of mental health disorders in YP can reach up to 13.5%, with anxiety and depression leading as the most common presentations (Polanczyk et al., 2015; Cohen et al., 2018). In England, recent survey data reported that one in eight 5 to 19 year olds had at least one mental health disorder and one in twenty met criteria for two or more mental health diagnoses (NHS Digital, 2019). Consequently, treatment options including psychotherapy and more recently digital interventions are being incorporated to support YP and families (Das et al., 2016; Liverpool et al., 2020). Although there is some evidence suggesting the effectiveness and efficacy of these interventions, many studies report limitations such as low engagement, non-adherence, and dropout from treatment, having implications for premature endings and case closure (Kazdin et al., 1994; Kazdin, 1997; Gopalan et al., 2010).

### Proposed Explanations and Categorisations of Case Closure

Premature termination, defined as when a “client has left therapy before obtaining a requisite level of improvement or completing therapy goals” (Hatchett and Park, 2003, p. 226) is a significant and widespread problem in the field of mental health (Barrett et al., 2008). Up to 50% of clients discontinue psychological services prematurely (Barrett et al., 2008) which undermines the potential benefits of treatment and reduces the cost-effectiveness of these interventions (Westmacott et al., 2010). Several studies examining potential variables associated with this phenomenon (i.e., client, therapist, and treatment) have been conducted (Wierzbicki and Pekarik, 1993; Garfield, 1994; Sales, 2003; Clarkin and Levy, 2004), but results are largely inconsistent due to the plethora of terms that are often interchangeably used (i.e., attrition, dropout, early termination, pre-mature termination, early withdrawal, among others) (Wierzbicki and Pekarik, 1993) and the methods adopted to operationalize these constructs (Swift et al., 2009). To illustrate, main categorizations of dropout usually include *duration* of the therapy (i.e., when the YP in a study terminates treatment before the pre-defined cut off) and *therapist judgment* of whether the treatment termination is a dropout. However, it is often difficult for therapists to detect how clients are responding to therapy (Hannan et al., 2005). Nonetheless, therapists' and the YPs' or carers' assumptions about treatment goals and expectations may differ (Barrett et al., 2008), leading to non-mutually agreed decisions (de Haan et al., 2013). In fact, whether or not criteria for “clinical improvement” or recovery have been met, clients may prematurely end treatment because the necessary gains in functioning have been obtained *prior* to the end of a set number of sessions, or because they may want to try other interventions on their own, outside of treatment.

On the other hand, clients may recognize a lack of improvement and believe that additional sessions will not be helpful, another perspective that can also be difficult to detect during therapy (Lambert et al., 2005). Further, the type of treatment a client receives also influences rates of non-mutually agreed endings in therapy (Barrett et al., 2008). Treatments involving both medications and therapy in the extant literature have consistently shown lower rates of attrition than either medication or therapy alone (Arnow et al., 2007). Another reason for dropout includes the YP's diagnosis (Westmacott et al., 2010). Researchers reported higher rates of attrition among clients with more severe diagnoses (i.e., externalizing problems) and more complex diagnostic pictures (i.e., comorbidity) (Thormählen et al., 2003). There is also some evidence showing that external factors may also influence YP's use of health care services or constitute barriers to continuing treatment. Such factors include difficulties in finding mental health services, cost for services, degree of family involvement, and social support networks. Beyond that, practical issues such as greater distance traveled, scheduling conflicts, and long waiting lists can negatively influence community perception of the mental health services resulting in earlier dropout from care (Westmacott et al., 2010). Therefore, a need-based definition is a valuable method for categorizing treatment dropouts and mitigates disadvantages of existing definitions of dropout (Dossett and Reid, 2019).

### Current State of Associations With Case Closure

Demographic data such as belonging to an ethnic minority (de Haan et al., 2018) or lower socioeconomic status group (de Haan et al., 2014), having a younger mother, and living in a single-parent household (de Haan et al., 2013) are social and family variables that increase the likelihood of dropping out of treatment. Despite this, variables related to the treatment itself and those related to the therapist were also found to be overall stronger dropout predictors than the pre-treatment child and family or parent/carer variables. Specifically, dropout increases when adolescents experience lower quality of the therapeutic relationship, lower perceived relevance of treatment, more treatment participation barriers, and more stressors (Carter, 1995; Garcia and Weisz, 2002). Significant predictors of dropout are also the adolescent's experience of their therapist as being directive, controlling, and confronting; the therapist not showing care and concern; and dissatisfaction with the focus of therapy (Jethwa et al., 2019). More cancellations or no-shows over the course of the treatment have also been consistently found as a reliable predictor of dropout (Kazdin et al., 1994; Chasson et al., 2008; de Haan et al., 2013). Emerging evidence highlights the importance of including cultural understanding and adoption in the therapeutic relationship in order to retain YP in mental health settings (Yeh et al., 1994; Carter, 1995; Cunningham et al., 2002; Lau, 2006; Huey and Polo, 2008; Miller et al., 2008; Bibi et al., 2017).

Nonetheless, treatment dropout is often regarded as a negative outcome in therapy. A mixed-method study that explored YP's reasons for dropout from treatment highlighted that nearly one third of the sample indicated they had received satisfactory treatment or experienced symptom improvement (O'Keeffe et al., 2019a). Therefore, when clients end treatment non-mutually, their therapists are often not aware whether their clients were (dis)satisfied with the therapy (Westmacott et al., 2010). However, therapists of YP who dropped out due to symptom improvement reported they were not clinically concerned about this group of dropouts. This indicates that treatment terminations following clients who benefitted from therapy may not yet meaningfully be accounted for in existing explanatory models of dropout from treatment (O'Keeffe et al., 2019a).

### The Current Study

There is a growing interest in improving outcomes for YP accessing mental health services, with the main focus thus far being on improving symptoms and aiming for “recovery”. However, other outcomes may also be important, and in particular, whether YP and therapist mutually agree with the end of treatment. This line of reasoning may have implications for the evaluation of outcomes at the case level and service level, including accuracy of data and effective use of costs to the National Health Services (Mental Health Taskforce to the NHS in England, 2016). Therefore, it is important to broaden our understanding of the influence of demographics, referral process, and symptom improvements on case closure. Evidence that non mutual case closure may not necessarily be a problem may reflect more self-efficacy, competence, self-rated improvement, and autonomy among YP and their carers (Simon et al., 2012; O'Keeffe et al., 2019b). Despite this wealth of knowledge, we are yet to fully understand if symptom improvement is an indicator of a “good” outcome.

In this vein, the present study aimed to examine whether levels of meaningful improvement in symptoms were associated with reasons for ending treatment, using multilevel multinomial regression analysis controlling for age, sex, ethnicity, and referral source. We hypothesized that youths whose problems meaningfully improved were more likely to mutually agree to end treatment.

## Methods

### Participants and Procedure

Three datasets held by the Child Outcomes Research Consortium on children and young people (0-25 years old) who accessed mental health services in the United Kingdom (UK) between 2002 and 2019 were merged (Costa da Silva et al., submitted). The data corpus was collected by clinicians and service administrators from YP mental health services across England, including those participating in a programme offered by the National Health Services to implement evidence-based practice between 2011 and 2015 (Fonagy et al., 2017). From this merged dataset, cases were included in the present analysis if: (a) the child or young person was aged 6-25 years to reflect the age range that the included measures could be self-reported, (b) the case was closed, (c) there was at least one paired outcome measure completed at time 1 and time 2, and (d) there was a reason for case closure. This resulted in a final dataset of *N* = 8,995 episodes of care (i.e., independent observations) [Female = 5,469, 61%; meanAge = 13.66 (SD = 2.87) years]. Detailed demographic characteristics are shown in Table 1.

**Table 1:** Descriptive statistics for all study variables.

	***n***	**%**	
**Demographics**			
Female	5,469	60.80	
Male	3,526	39.20	
Age (*M, SD*, range)	13.66	2.87	6-25
*Ethnicity*			
Asian	394	4.38	
Black	465	5.17	
Mixed-race	415	4.61	
Not reported	1,078	11.98	
Other ethnic group	226	2.51	
White British	6,026	66.99	
White other	391	4.35	
**Referral source**			
Primary care	3,265	36.3	
Self-referral	584	6.49	
Education	1,388	15.43	
Social care/ youth justice	372	4.14	
Child health	346	3.85	
Mental health	1,545	17.18	
Other	461	5.13	
Missing	1,034	11.5	
**Case closure reason**			
Mutual agreement	6,519	72.47	
Non-attendance	1,082	12.03	
Referral	545	6.06	
Other	849	9.44	
**Meaningful change**			
Improved	3,943	43.84	
No change	4,232	47.05	
Deteriorated	820	9.12	

*N = 8,995 from 68 services with 2–1,274 per service*.

### Ethical Considerations

The present analysis involved secondary analysis of anonymised administrative data and therefore, an ethical review was not required (Tripathy, 2013).

### Measures

#### Demographic and Clinical Characteristics

Age, gender, and ethnicity were recorded by services as part of routine data recording. Ethnicity was captured using the categories from the 2001 Census (Office for National Statistics, 2019) and was generally based on self-report by the parent/carer or the young person. These were grouped for analysis as follows: White British (as the ethnic majority group), White Other (including Irish and Other White background), mixed-race (including Mixed White and Black Caribbean, Mixed White and Black African, Mixed White and Asian, and any other mixed background), Asian (including Indian, Pakistani, Bangladeshi, and Other), Black or Black British (including Caribbean, African, and Other), other ethnic groups (including Chinese and Other), and not stated. As used in previous research, referral source was recorded by services using 30 indicators, which were grouped into nine study variables for the present analysis (Edbrooke-Childs and Patalay, 2019). In the main analysis, referral from primary care was selected as the reference category as it was the largest group.

#### Symptom Improvement

To measure symptom improvement, meaningful change according to self-reported measures was used. Meaningful change is the current analytic approach used by policy in England to examine national administrative data from child and adolescent mental health services. As we report elsewhere (Costa da Silva et al., submitted), meaningful change consisted of reliable change in standardized measures, or change more than would be expected solely from measurement error, and clinically important change in idiographic measures. For each completed measure at time 1 and time 2, it is therefore possible to improve, not change, or deteriorate according to reliable or clinically important change. YP were then classified as: (a) meaningfully improved if they met the criteria for improvement on at least one completed measure at time 1 and time 2 and did not deteriorate on any other measure, (b) not meaningfully changed if no completed measure at time 1 or time 2 met the criteria for reliable or clinically important change, or (c) meaningfully deteriorated if they met the criteria for deterioration on any completed measure at time 1 and time 2.

#### Case Closure Reason

Case closure reason was recorded by services and grouped into four categories for the present analysis: mutual agreement, non-mutual agreement, transfer, and other.

### Statistical Analysis

To examine whether YP who meaningfully improved were more likely to mutually agree to end treatment, accounting for the nesting of episodes of care in services and controlling for age, gender, ethnicity, and referral source, multilevel multinomial logistic regressions were conducted in STATA 16 (StataCorp., 2019). Three preparatory models were estimated. In *Model 0* (null model) the variance explained in case closure reason at the service-level was examined and no predictors were added. The intraclass correlation coefficient was 45%, indicating that there was significant service-level variation in case closure reason and confirming that multilevel modeling was the appropriate statistical approach. In *Model 1*, demographic characteristics were added: male; grand-mean-centered age; and ethnicity with the White British group as the reference category as it was the largest group. In *Model 2*, referral source was added with primary care as the reference category. In the final model, meaningful change was added with no change selected as the reference category as it was the largest group. The likelihood ratio test was used to compare successive models, which were significant, and all variables were therefore retained in the final model. In particular, the likelihood ratio test was significant for the final model compared to Model 2: χ^2^(6) = 111.3, *p* < 0.001.

## Results

The results of the final model are shown in Table 2. Compared to girls, boys were less likely to have case closure due to non-mutual agreement than case closure due to mutual agreement. Compared to younger YP, older YP were more likely to have case closure due to non-mutual agreement and transfer than case closure due to mutual agreement. Compared to White British YP, Black or Black British YP, mixed-race YP, and those from other White backgrounds were more likely to have case closure due to non-mutual agreement than case closure due to mutual agreement. Compared to White British YP, mixed-race YP were more likely to have case closure due to transfer than case closure due to mutual agreement. In contrast, compared to White British YP, YP with not reported ethnic backgrounds were less likely to have case closure reason due to transfer than case closure due to mutual agreement. Compared to White British YP, Asian YP, mixed-race YP, and YP with “other” ethnic backgrounds were less likely to have case closure due to other reasons than case closure due to mutual agreement. Compared to White British YP, YP with not reported ethnic backgrounds were more likely to have case closure due to other reasons than case closure due to mutual agreement.

**Table 2:** Multilevel multinomial regression with demographics, referral source, and meaningful improvement predicting case closure reason.

	**Non-mutual vs. mutual agreement**				**Transfer vs. mutual agreement**				**Other reason vs. mutual agreement**			
	**OR**	***p*-value**	**95% CI**		**OR**	***p*-value**	**95% CI**		**OR**	***p*-value**	**95% CI**	
**Demographics**												
Male vs. female	**0.83**	0.01500	0.71	0.96	1.14	0.18400	0.94	1.38	1	0.98200	0.85	1.18
Age	**1.09**	0.00000	1.06	1.12	**1.12**	0.00000	1.08	1.16	1.02	0.24800	0.99	1.05
*Ethnicity*												
Asian vs. WB	1.01	0.95400	0.71	1.43	1.15	0.51700	0.75	1.75	**0.61**	0.02700	0.39	0.94
Black vs. WB	**1.38**	0.03500	1.02	1.85	1.02	0.90900	0.68	1.55	0.74	0.13500	0.50	1.10
Mixed-race vs. WB	**1.48**	0.01100	1.09	2.01	**1.6**	0.01600	1.09	2.33	**0.6**	0.02400	0.38	0.93
Not reported vs. WB	0.92	0.48200	0.72	1.17	**0.57**	0.00200	0.40	0.81	**1.29**	0.04000	1.01	1.65
Other ethnic group vs. WB	1.07	0.76500	0.69	1.66	1.07	0.81000	0.62	1.83	**0.28**	0.00100	0.13	0.60
White other vs. WB	**1.4**	0.03800	1.02	1.91	0.95	0.81800	0.61	1.48	0.86	0.44400	0.59	1.26
**Referral source**												
Self-referral vs. pri. care	1.36	0.06700	0.98	1.90	**0.28**	0.00000	0.14	0.54	**0.48**	0.00100	0.30	0.75
Education vs. pri. care	0.87	0.33500	0.66	1.15	**0.57**	0.00200	0.40	0.81	0.89	0.39000	0.67	1.17
Social care/ youth justice vs. pri. care	**1.45**	0.04100	1.01	2.06	**1.62**	0.02000	1.08	2.43	0.82	0.37500	0.53	1.27
Child health vs. pri. care	1.25	0.28000	0.83	1.88	**1.59**	0.04300	1.02	2.50	0.92	0.70600	0.59	1.43
Mental health vs. pri. care	1.14	0.26000	0.91	1.42	1.12	0.39100	0.86	1.46	**1.25**	0.04700	1.00	1.56
Other vs. primary care	**1.6**	0.01200	1.11	2.30	**0.49**	0.03500	0.25	0.95	0.89	0.60900	0.57	1.39
Missing vs. primary care	**1.8**	0.00000	1.39	2.34	1.16	0.35500	0.84	1.61	**0.59**	0.00100	0.43	0.81
**Meaningful change**												
Improved vs. no change	**0.58**	0.00000	0.50	0.68	**0.61**	0.00000	0.49	0.74	**0.59**	0.00000	0.50	0.70
Deteriorated vs. no change	1.25	0.06000	0.99	1.57	**1.38**	0.02900	1.03	1.83	**0.62**	0.00200	0.46	0.84

*N = 8,995 from 68 services with 2-1,274 per service. OR = odds ratio. CI = 95% Confidence Interval. WB = White British. Odds ratios in bold are significant at least at the p < 0.05 level*.

Compared to YP referred by primary care, YP referred through social care/ youth justice, other sources, and with missing referral source were more likely to have case closure due to non-mutual agreement than case closure due to mutual agreement. Compared to YP referred by primary care, YP referred by self-referral, education, or other sources were less likely to have case closure due to transfer than case closure due to mutual agreement. In contrast, compared to YP referred by primary care, YP referred by social care/ youth justice or child health were more likely to have case closure due to transfer than case closure due to mutual agreement. Compared to YP referred by primary care, YP referred by mental health services were more likely to have case closure due to other reasons, and YP referred by self-referral or with missing referral source were less likely to have case closure due to other reasons, than case closure due to mutual agreement.

Compared to YP who did not meaningfully change in symptoms, YP who meaningfully improved in symptoms were less likely to have case closure due to non-mutual agreement, transfer, and other reasons than case closure due to mutual agreement. Compared to YP who did not meaningfully change in symptoms, YP who meaningfully deteriorated in symptoms were more likely to have case closure due to transfer, and were less likely to have case closure due to other reasons, than case closure due to mutual agreement.

## Discussion

To better understand symptom improvement as an indicator of a good outcome of accessing YP mental health services, this study examined whether levels of meaningful improvement were associated with reasons for ending treatment. Multilevel multinomial regression analyses were conducted controlling for age, gender, ethnicity, and referral source. As hypothesized, the results indicated that YP whose problems meaningfully improved were more likely to mutually agree to end treatment.

Our results are consistent with previous studies showing improved mental health to be associated with treatment completion when compared to YP who prematurely ended treatment (Kazdin et al., 1994; Chasson et al., 2008; de Haan et al., 2013). Nevertheless, the present study builds on the extant literature as it is the largest study on symptom improvement and reasons for case closure. Moreover, this study used an advanced statistical approach to account for service-level variation. This study also uses the latest approach to measuring symptom improvement using meaningful change.

A possible explanation could be that YP who do not experience improvement are more likely to go on to access adult care or other specialist services, which this study highlighted. This is consistent with studies in adult mental health services (Westmacott et al., 2010; Bartholomew et al., 2019). These findings may also be attributed to treatment engagement which can be affected by diagnostic agreement (Jensen-Doss and Weisz, 2008) and shared treatment decision-making experiences in YP mental health services (Edbrooke-Childs et al., 2015). Further, existing research suggests the most common reason for non-mutual treatment endings in YP therapeutic settings was a therapeutic relationship disconnect (Carter, 1995; Garcia and Weisz, 2002). Although the current findings show significant associations between meaningful change and mutual agreement to end treatment, a recent study found no significant evidence linking YP depressive symptoms to mutual agreement on treatment ending (O'Keeffe et al., 2019b). This inconsistency may warrant further investigations if we are to generalize findings across symptom type, treatment type, and the level of impact the psychosocial difficulties may have on the YP and their families.

The current findings also reflect further potential disparities and child mental health inequalities in the UK (Fairchild, 2019). In comparison to White British YP, Black or Black British YP, mixed-race YP, and YP from other White ethnic backgrounds were more likely to have case closure due to non-mutual agreement than have case closure due to mutual agreement. It is likely that such connections exist highlighting associations such as ethnic minority groups being more likely to access YP mental health services through non-voluntary routes, for example, social care/ youth justice (Edbrooke-Childs and Patalay, 2019). This is important because the current findings suggest that YP who access services through more compulsory sources, such as social care/ youth justice, were more likely to have case closure reason due to non-mutual agreement and transfer than case closure due to mutual agreement. These findings may possibly support previous research outlining socio-economic disadvantages as a predictor of dropout from treatment, which include factors such as a lack of transportation and childcare (Kazdin et al., 1994; Kazdin, 1997; de Haan et al., 2013). However, it is still unclear which mediating factors may influence these findings as previous research fails to associate these demographic factors with treatment outcome and ending (O'Keeffe et al., 2019a).

Yet, there is some suggestion that the interface between difficulties and the type of intervention may be the effective element in YP retention (Baruch et al., 1998; Johnson et al., 2009). This poses a question whether relevant and effective treatments are being offered to YP with the most severe and complex needs.

Whilst the finding that YP who achieve meaningful improvement are likely to end treatment on mutual terms, there are also methodological and outcome tracking considerations here. Previous research suggests that clients may disengage from treatment when they have reached a level of “recovery” that is important to them (Hynan, 1990; McKenna and Todd, 1997; Todd et al., 2003). Therefore, there may be a discord between the outcomes of importance to the clinician and young person. If YP feel as though they have reached a level of recovery or improvement that is important to them, they may discontinue treatment regardless of how much progress they have made on a symptom-based measure. Thus, highlighting the importance of collecting a range of outcome information, and further highlighting the importance of shared decision-making.

### Implications

Although our findings suggest that YP who meaningfully improve are more likely to mutually agree to ending treatment, clinicians and researchers should consider that some YP may non-mutually end treatment if they self-assess as having sufficiently improved. This speaks in favor of ongoing evaluations of treatment goals and progress tracking. In light of the previous literature, it is also important to note that families with YP diagnosed with specific difficulties, having additional complexities, or experiencing external variables such as deprivation are more vulnerable to non-mutually end treatment. Therefore, researchers, clinicians, families, YP, and decision-makers should continue to work together to develop tailored service level programmes and individual interventions to ensure underrepresented and underserved families are reached. For example, the finding that YP from non-White British ethnic groups are more likely to drop out of treatment highlights the importance of reaching these groups. This includes considering the referral routes and types of interventions offered, including consideration of community-based interventions, which may widen reach and increase retention for the identified groups.

One area that was not possible to investigate in the present study is the parent/carer perspective, given the significant role parents and carers have in YP retention in mental health settings (Weisz et al., 1987; Garcia and Weisz, 2002). Future research should explore this, as parent/carer views may differ from those of the clinician and the young person. It is also important to continue research into the use of digital interventions. With growing interest in this area, through web-based appointment systems and texting to mobile phones, we may be able to better capture reasons for treatment dropouts and facilitate non-face-to-face support for YP. Further qualitative and quantitative studies are also welcomed to explore YP's own descriptions of good outcomes and treatment ending to triangulate or further develop our current descriptions.

### Strengths and Limitations

A major strength of this study is the inclusion of a large sample size. Moreover, we investigated the factors associated with case closure and YP's mental health using multilevel modeling, a method that was able to account for of individual and service-level variation. However, these results should be interpreted in the context of several limitations. The large majority of the participants were female and identified as White-British ethnicity thus preventing us from making predictions on the impact that cultural variations may have on the study's findings. Moreover, the specific problems presented by YP may have influenced the study outcome, but we were unable to account for this in the present investigation.

Another limitation of the study relates to the numerous ways that dropout can be defined, bringing challenges to the ability to compare results between studies (Barrett et al., 2008; de Haan et al., 2013). Reliability of the study's results is also affected by the absence of detailed information on professionals' reasons for case closure and the lack of qualitative data from YP or parents/carers in order to provide a deeper understanding of the current sample. In addition, the unavailability of follow-up data prevents drawing conclusions about the efficacy and effectiveness of the intervention – therefore on the extent to which clients' decision to discontinue the therapy due to perceived improvements or dissatisfaction is supported by trends in symptoms or clinical outcomes. Without a randomized controlled design, inferences about causation, of symptom improvement and reason for case closure, cannot be made. Another constraint identified was the reliance on routine pre-collected data, resulting in less flexibility to include explanatory variables of interest, such as the parent/carer perspective. Although this may compromise the rigorous empirical research standards and cause-effect relationships, this method has the benefit of allowing us to investigate variables without additional research participation burden to YP (Mansfield et al., 2020).

## Conclusion

Symptom improvement continues to be an important indicator assess a good outcome that in turn determines treatment ending. The findings of the current study provide support for this approach indicating that YP with improvements are more likely to mutually agree to ending treatment. However, it is noted that symptom improvement should be evaluated alongside other aspects of the YP's life situation. Although further research is needed to fully conceptualize and understand non-mutually agreed endings (e.g., dropout), the current findings contribute to informing evidence-based practice.

## Data Availability Statement

The data analyzed in this study is subject to the following licenses/restrictions: Request for administrative data can be made available upon request from the corresponding author. Requests to access these datasets should be directed to julian.childs@annafreud.org.

## Ethics Statement

As this was a secondary analysis of anonymised data, ethical review and approval was not required for the study on human participants in accordance with the local legislation and institutional requirements. Written informed consent from the participants' legal guardian/next of kin was not required to participate in the present analysis in accordance with the national legislation and the institutional requirements.

## Author Contributions

JE-C and JJ conceived of the study. JE-C lead the data analysis and methods and results writing, to which TB, CS, and JJ contributed. CPM, GP, CS, and NF reviewed the literature and wrote the introduction. RU, AČ, and SL wrote the discussion. All authors contributed to the analysis decisions, reviewed and edited the manuscript, and approved the final version.

## Conflict of Interest

TB reports other funding from NHS England & NHS Improvement, outside the submitted work. JE-C reports grants from NHS England & NHS Improvement, outside the submitted work, and he was involved in the programme of service transformation that some of the data in the present manuscript draws on. The remaining authors declare that the research was conducted in the absence of any commercial or financial relationships that could be construed as a potential conflict of interest.

## Abbreviations

YP: Young people.

## References

[B1] Arnow B. A. Blasey C. Manber R. Constantino M. J. Markowitz J. C. Klein D. N.. (2007). Dropouts versus completers among chronically depressed outpatients. J. Affect. Disord. 97, 197–202. 10.1016/j.jad.2006.06.017 16857266

[B2] Barrett M. S. Chua W. J. Crits-Christoph P. Gibbons M. B. Thompson D. (2008). Early withdrawal from mental health treatment: implications for psychotherapy practice. Psychotherapy 45, 247–267. 10.1037/0033-3204.45.2.247 19838318 PMC2762228

[B3] Bartholomew T. T. Lockard A. J. Folger S. F. Low B. E. Poet A. D. Scofield B. E.. (2019). Symptom reduction and termination: client change and therapist identified reasons for saying goodbye. Couns. Psychol. Q. 32, 81–99. 10.1080/09515070.2017.1367272

[B4] Baruch G. Gerber A. Fearon P. (1998). Adolescents who drop out of psychotherapy at a community-based psychotherapy centre: a preliminary investigation of the characteristics of early drop-outs, late drop-outs and those who continue treatment. Br. J. Med. Psychol. 71, 233–245. 10.1111/j.2044-8341.1998.tb00988.x 9733419

[B5] Bibi F. Millar H. Holland A. (2017). The role of cultural factors in engagement and change in multisystemic therapy (MST). J. Fam. Ther. 39, 243–263. 10.1111/1467-6427.12134

[B6] Camilletti E. (2018). Realizing an Enabling Environment for Adolescent Well-Being: An Inventory of Laws and Policies for Adolescents in South Asia. UNICEF Office of Research - Innocenti.

[B7] Carter R. (1995). The Influence of Race and Racial Identity in Psychotherapy: Toward a Racially Inclusive Model. New Jersey, NJ: John Wiley and Sons.

[B8] Chasson G. S. Vincent J. P. Harris G. E. (2008). The use of symptom severity measured just before termination to predict child treatment dropout. J. Clin. Psychol. 64, 891–904. 10.1002/jclp.20494 18459120

[B9] Clarkin J. F. Levy K. N. (2004). The influence of client variables on psychotherapy, in Bergin and Garfield's Handbook of Psychotherapy and Behavior Change, 5th ed, ed Lambert M. J. (New York, NY: Wiley and Sons), 194–226.

[B10] Cohen J. R. Andrews A. R. Davis M. M. Rudolph K. D. (2018). Anxiety and depression during childhood and adolescence: testing theoretical models of continuity and discontinuity. J. Abnorm. Child Psychol. 46, 1295–1308. 10.1007/s10802-017-0370-x 29256025 PMC6008170

[B11] Cunningham P. B. Foster S. L. Henggeler S. W. (2002). The elusive concept of cultural competence. Child. Serv. 5, 231–243. 10.1207/S15326918CS0503_7

[B12] Das J. K. Salam R. A. Lassi Z. S. Khan M. N. Mahmood W. Patel V.. (2016). Interventions for adolescent mental health: an overview of systematic reviews. J. Adolesc. Health 59, 49–60. 10.1016/j.jadohealth.2016.06.020 27664596 PMC5026677

[B13] de Haan A. M. Boon A. E. de Jong J. T. V. M. Hoeve M. Vermeiren R. R. J. M. (2013). A meta-analytic review on treatment dropout in child and adolescent outpatient mental health care. Clin. Psychol. Rev. 33, 698–711. 10.1016/j.cpr.2013.04.005 23742782

[B14] de Haan A. M. Boon A. E. de Jong J. T. V. M. Vermeiren R. R. J. M. (2018). A review of mental health treatment dropout by ethnic minority youth. Transcult. Psychiatry 55, 3–30. 10.1177/1363461517731702 29035137

[B15] de Haan A. M. Boon A. E. Vermeiren R. R. J. M. Hoeve M. de Jong J. T. V. M. (2014). Ethnic background, socioeconomic status, and problem severity as dropout risk factors in psychotherapy with youth. Child Youth Care Forum. 44, 1–16. 10.1007/s10566-014-9266-x

[B16] Dossett K. W. Reid G. J. (2019). Defining dropout from children's mental health services: a novel need-based definition. J. Child Fam. Stud. 29, 2028–2038. 10.1007/s10826-019-01631-1

[B17] Edbrooke-Childs J. Jacob J. Argent R. Patalay P. Deighton J. Wolpert M. (2015). The relationship between child- and parent-reported shared decision making and child-, parent-, and clinician-reported treatment outcome in routinely collected child mental health services data. Clin. Child Psychol. Psychiatry 21, 324–338. 10.1177/1359104515591226 26104790

[B18] Edbrooke-Childs J. Patalay P. (2019). Ethnic differences in referral routes to youth mental health services. J. Am. Acad. Child Adolesc. Psychiatry 58, 368–375.e1. 10.1016/j.jaac.2018.07.906 30768415

[B19] Fairchild G. (2019). Mind the gap: evidence that child mental health inequalities are increasing in the UK. Eur. Child Adolesc. Psychiatry 28, 1415–1416. 10.1007/s00787-019-01418-1 31605207

[B20] Fonagy P. Pugh K. O'Herlihy A. (2017). The children and young people's improving access to psychological therapies (CYP IAPT) programme in England. Child Psychol. Psychiatry 429–35. 10.1002/9781119170235.ch48

[B21] Garcia J. A. Weisz J. R. (2002). When youth mental health care stops: therapeutic relationship problems and other reasons for ending youth outpatient treatment. J. Consult. Clin. Psychol. 70, 439–443. 10.1037/0022-006X.70.2.439 11952203

[B22] Garfield S. L. (1994). Research on client variables in psychotherapy, in Handbook of Psychotherapy and Behavior Change, eds Bergin A. E. Garfield S. L. (New Jersey, NJ: John Wiley and Sons), 190–228.

[B23] Gopalan G. Goldstein L. Klingenstein K. Carolyn Sicher Psy D. Blake C. McKay M. M. (2010). Engaging families into child mental health treatment: Updates and special considerations. J. Can. Acad. Child Adolesc. Psychiatry 19, 182–196. 20842273 PMC2938751

[B24] Hannan C. Lambert M. J. Harmon C. Nielsen S. L. Smart D. W. Shimokawa K.. (2005). A lab test and algorithms for identifying clients at risk for treatment failure. J. Clin. Psychol. 61, 155–163. 10.1002/jclp.20108 15609357

[B25] Hatchett G. T. Park H. L. (2003). Comparison of four operational definitions of premature termination. Psychotherapy 40, 226–231. 10.1037/0033-3204.40.3.226

[B26] Huey S. J. Polo A. J. (2008). Evidence-based psychosocial treatments for ethnic minority youth. J. Clin. Child Adolesc. Psychol. 37, 262–301. 10.1080/15374410701820174 18444061 PMC2413000

[B27] Hynan D. J. (1990). Client reasons and experiences in treatment that influence termination of psychotherapy. J. Clin. Psychol. 46, 891–895. 10.1002/1097-4679(199011)46:6<891::AID-JCLP2270460631>3.0.CO;2-8 2286687

[B28] Jensen-Doss A. Weisz J. R. (2008). Diagnostic agreement predicts treatment process and outcomes in youth mental health clinics. J. Consult. Clin. Psychol. 76, 711–722. 10.1037/0022-006X.76.5.711 18837589

[B29] Jethwa J. Glorney E. Adhyaru J. Lawson A. (2019). A grounded theory of multisystemic therapist roles in achieving positive outcomes for young people and families. J. Fam. Ther. 43, 1–19. 10.1111/1467-6427.12287

[B30] Johnson E. Mellor D. Brann P. (2009). Factors associated with dropout and diagnosis in child and adolescent mental health services. Aust. N. Z. J. Psychiatry 43, 431–437. 10.1080/00048670902817687 19373704

[B31] Kazdin A. (1997). Practitioner review: psychosocial treatments for conduct disorder in children. J. Child Psychol. Psychiatry Allied Discip. 38, 161–178. 10.1111/j.1469-7610.1997.tb01851.x 9232463

[B32] Kazdin A. Mazurick J. Siegel T. (1994). Treatment outcome among children with externalizing disorder who terminate prematurely versus those who complete psychotherapy. J. Am. Acad. Child Adolesc. Psychiatry 33, 549–557. 10.1097/00004583-199405000-00013 8005908

[B33] Lambert M. Harmon C. Slade K. Whipple J. Hawkins E. (2005). Providing feedback to psychotherapists on their patients' progress: clinical results and practice suggestions. J. Clin. Psychol. 61, 165–174. 10.1002/jclp.20113 15609358

[B34] Lau A. S. (2006). Making the case for selective and directed cultural adaptations of evidence-based treatments: examples from parent training. Clin. Psychol. Sci. Pract. 13, 295–310. 10.1111/j.1468-2850.2006.00042.x

[B35] Liverpool S. Mota C. P. Sales C. M. D. Cuš A. Carletto S. Hancheva C.. (2020). Engaging children and young people in digital mental health interventions: systematic review of modes of delivery, facilitators, and barriers. J. Med. Internet Res. 22:e16317. 10.2196/16317 32442160 PMC7381028

[B36] Mansfield K. L. Gallacher J. E. Mourby M. Fazel M. (2020). Five models for child and adolescent data linkage in the UK: a review of existing and proposed methods. Evid. Based Ment. Health, 23, 39–44. 10.1136/ebmental-2019-300140 32046992 PMC7034351

[B37] McKenna P. A. Todd D. M. (1997). Longitudinal utilization of mental health services: a timeline method, nine retrospective accounts, and a preliminary conceptualization. Psychother. Res. 7, 383–395. 10.1080/10503309712331332093

[B38] Mental Health Taskforce to the NHS in England (2016). The Five Year Forward View for Mental Health. The Mental Health Taskforce. Available online at: https://www.england.nhs.uk/wp-content/uploads/2016/02/Mental-Health-Taskforce-FYFVfinal.pdf (accessed Febraury 1, 2021).

[B39] Miller L. M. Southam-Gerow M. A. Allin R. B. (2008). Who stays in treatment? Child and family predictors of youth client retention in a public mental health agency. Child Youth Care Forum 37, 153–170. 10.1007/s10566-008-9058-2 19774098 PMC2747739

[B40] NHS Digital (2019). Waiting Times for Children and Young People's Mental Health Services, 2018 - 2019 Additional Statistics. Available online at: https://digital.nhs.uk/data-and-information/find-data-and-publications/supplementary-information/2019-supplementary-information-files/waiting-times-for-children-and-young-peoples-mental-health-services-2018–2019-additional-statistics (accessed Febraury 1, 2021).

[B41] Office for National Statistics (2019). Office for National Statistics. UNEM01 SA: Unemployment by Age and Duration (Seasonally Adjusted).

[B42] O'Keeffe S. Martin P. Goodyer I. M. Kelvin R. Dubicka B. Reynolds S.. (2019a). Prognostic implications for adolescents with depression who drop out of psychological treatment during a randomized controlled trial. J. Am. Acad. Child Adolesc. Psychiatry 58, 983–992. 10.1016/j.jaac.2018.11.019 30946974

[B43] O'Keeffe S. Martin P. Target M. Midgley N. (2019b). I just stopped going”: a mixed methods investigation into types of therapy dropout in adolescents with depression. Front. Psychol. 10:75. 10.3389/fpsyg.2019.00075 30804827 PMC6370696

[B44] Polanczyk G. V Salum G. A. Sugaya L. S. Caye A. Rohde L. A. (2015). Annual research review: a meta-analysis of the worldwide prevalence of mental disorders in children and adolescents. J. Child Psychol. Psychiatry Allied Discip. 56, 345–365. 10.1111/jcpp.12381 25649325

[B45] Sales C. M. D. (2003). Understanding prior dropout in psychotherapy. Int. J. Psychol. Psychol. Ther. 3, 81–90.

[B46] Simon G. E. Imel Z. E. Ludman E. J. Steinfeld B. J. (2012). Is dropout after a first psychotherapy visit always a bad outcome? Psychiatric Serv. 63, 705–707. 10.1176/appi.ps.201100309 22752034 PMC3708593

[B47] StataCorp. (2019). Stata Statistical Software: Release 16. College Station, TX: StataCorp LP.

[B48] Swift J. K. Callahan J. Levine J. C. (2009). Using clinically significant change to identify premature termination. Psychotherapy 46, 328–335. 10.1037/a0017003 22122724

[B49] Thormählen B. Weinryb R. M. Norén K. Vinnars B. Bågedahl-Strindlund M. Barber J. P. (2003). Patient factors predicting dropout from supportive-expressive psychotherapy for patients with personality disorders. Psychother. Res. 13, 493–509. 10.1093/ptr/kpg039 21827258

[B50] Todd D. M. Deane F. P. Bragdon R. A. (2003). Client and therapist reasons for termination: a conceptualization and preliminary validation. J. Clin. Psychol. 59, 133–147. 10.1002/jclp.10123 12508337

[B51] Tripathy J. P. (2013). Secondary data analysis: ethical issues and challenges. Iran. J. Public Health 42, 1478–1479. 26060652 PMC4441947

[B52] Weisz J. R. Weiss B. Langmeyer D. B. (1987). Giving up on child psychotherapy: who drops out? J. Consult. Clin. Psychol. 55, 916–918. 10.1037/0022-006X.55.6.916 3693659

[B53] Westmacott R. Hunsley J. Best M. Rumstein-Mckean O. Schindler D. (2010). Client and therapist views of contextual factors related to termination from psychotherapy: a comparison between unilateral and mutual terminators. Psychother. Res. 20, 423–435. 10.1080/10503301003645796 20560091 PMC2924572

[B54] Wierzbicki M. Pekarik G. (1993). A meta-analysis of psychotherapy dropout. Prof. Psychol. Res. Pract. 24, 190–195. 10.1037/0735-7028.24.2.190

[B55] Yeh M. Eastman K. Cheung M. K. (1994). Children and adolescents in community health centers: does the ethnicity or the language of the therapist matter? J. Commun. Psychol. 22, 153–163. 10.1002/1520-6629(199404)22:2<153::AID-JCOP2290220210>3.0.CO;2-R

